# Echocardiographic Techniques of Deformation Imaging in the Evaluation of Maternal Cardiovascular System in Patients with Complicated Pregnancies

**DOI:** 10.1155/2017/4139635

**Published:** 2017-08-22

**Authors:** Silvia Visentin, Chiara Palermo, Martina Camerin, Luciano Daliento, Denisa Muraru, Erich Cosmi, Luigi P. Badano

**Affiliations:** ^1^Department of Woman's and Child's Health, University of Padua, Padua, Italy; ^2^Department of Cardiac, Thoracic and Vascular Sciences, University of Padua, Padua, Italy

## Abstract

Cardiovascular diseases (CVD) represent the leading cause of maternal mortality and morbidity. Knowledge of CVD in women is constantly evolving and data are emerging that female-specific risk factors as complications of pregnancy are conditions associated with an increased risk for the long-term development of CVD. Echocardiography is a safe and effective imaging technique indicated in symptomatic or asymptomatic pregnant women with congenital heart diseases who require close monitoring of cardiac function. Deformation imaging is an echocardiographic technique used to assess myocardial function by measuring the actual deformation of the myocardium through the cardiac cycle. Speckle-tracking echocardiography (STE) is a two-dimensional (2D) technique which has been found to be more accurate than tissue Doppler to assess both left ventricular (LV) and right ventricular (RV) myocardial function. The use of 2D STE however might present some technical issues due to the tomographic nature of the technique and the motion in the three-dimensional space of the myocardial speckles. This has promoted the use of 3D STE to track the motion of the speckles in the 3D space. This review will focus on the clinical value of the new echocardiographic techniques of deformation imaging used to assess the maternal cardiovascular system in complicated pregnancies.

## 1. Epidemiology

Cardiovascular disease (CVD) remains the leading cause of maternal mortality, and maternal cardiac diseases are a major cause of nonobstetric morbidity, complicating 0.2 to 4% of pregnancies in industrialized countries. Older age at the first pregnancy and the increasing prevalence of hypertension, type II diabetes, and obesity represent the main risk factors for cardiovascular complications in pregnancy and postpartum [1]. Moreover, pregnant women with known heart diseases require multidisciplinary obstetric and medical management to assess maternal and fetal risks; modern ultrasonographic technologies are pivotal to allow these patients to reach the childbearing age. In industrialized countries, congenital heart diseases account for 75–82% of CVD in pregnancy, with shunt lesions predominating (20–65%), whereas in nonindustrialized countries the major cause is rheumatic valvular disease (56–89% of CVD in pregnancy). Finally, cardiomyopathies are rare, but they may potentially cause severe complications, and peripartum cardiomyopathy is the most common of them [2].

## 2. Physiologic Cardiovascular Modification during Pregnancy

During pregnancy, there are many physiological changes due to the increased metabolic demand of the mother-fetus couple, which requires an adequate uteroplacental circulation. Impairment of these mechanisms of adaptation can cause a fetal or maternal disease, such as growth retardation and preeclampsia, or unmask an underlying cardiac disease. Pregnancy is associated with an increase in heart rate, which starts in the first trimester, peaks in the third trimester (15–25% increase over the baseline heart rate), and returns to preconceptional values by 10 days postpartum. There is also a hormonally mediated increase in blood volume, red cells, and stroke volume (about 20–30%), although there are many difficulties in calculating it [3]. These changes lead to the increase in cardiac output by 30% in the first and second trimester, reaching sometimes 45% in a singleton pregnancy at 24 weeks. Whether or not cardiac output changes in the third trimester is currently debated. However, we know that it decreases rapidly in the first 2 weeks after the delivery, until 24 weeks postpartum. The increase in cardiac output seems to be due to increased stroke volume during early gestation, to increased heart rate later on [2–4]. Immediately after delivery, cardiac output further increases because of the decompression of the inferior vena cava and autotransfusion from the uterus.

Blood pressure typically decreases during normal gestation and it is usually 10 mmHg below baseline values in the second trimester. There is no agreement about systolic blood pressure change during pregnancy [2–8], but it appears likely that both diastolic and mean arterial blood pressure decrease from the early first trimester until 26–28 weeks and then they rise again towards the end of the pregnancy [3, 7–9]. Blood pressure changes are induced by a reduction in systemic vascular resistance [10]. The uteroplacental circulation and systemic vasodilatation contribute to the decline in vascular resistance, which is the steady component of ventricular afterload. Vascular resistance decreases in the early first trimester, presents its nadir in the middle of the second trimester, and returns close to prepregnancy levels within two weeks after delivery [11]. Associated with the decrease of vascular resistance, there is an increase in global arterial compliance (approximately 30%), which is the pulsatile component of ventricular afterload [2, 3]. The abovementioned hemodynamic changes can be considered the pathophysiological bases of cardiac remodeling during pregnancy [2, 12]. In fact, a 15% increase of left atrial diameter has been related to the increase in preload. It starts in the first 5 weeks and plateaus at 28–34 weeks' gestation. Similarly, the left ventricle (LV) increases its end-diastolic dimension by 7–12% beginning at 12 weeks and shows a plateau at 24–32 weeks [4]. Moreover, LV develops eccentric hypertrophy by increasing left ventricular wall thickness (15–25%) and mass (by 50%, mainly in the third trimester). These parameters of left ventricular remodeling may remain above normal limits until 6 months postpartum [10].

## 3. The Evaluation of Myocardial Function: Strain Imaging

Echocardiography is the most frequently used imaging technique to assess cardiac function and hemodynamics in CVD. Echocardiography allows a rapid assessment of systolic and diastolic function of cardiac chambers, regional wall motion, and valve anatomy and function [13]. Due to the safety of ultrasounds, the wide availability of the technique, and its portability and repeatability, echocardiography is very useful to assess the cardiovascular system of pregnant women with suspected or confirmed heart disease [2].

Deformation imaging is an echocardiographic technique used to assess myocardial function by measuring the actual change in length of the myocardium through the cardiac cycle. Echocardiography can evaluate myocardial deformation through two methods: the first, tissue velocity imaging (TDI), is a Doppler based method, whereas the second, speckle-tracking echocardiography (STE), is based on the analysis of conventional two-dimensional grayscale images.

Strain and strain rate, indices of myocardial deformation, can be obtained with both TDI and STE. Using color TDI, the SR is calculated as the velocity between 2 points along the myocardial wall (velocity gradient) normalized for the distance between the 2 points. TDI SR data can be integrated over time to obtain strain that is the most frequently used measure of myocardial deformation and measures the relative lengthening or shortening of myocardial fibers compared to baseline values. Since TDI is a Doppler based method and velocity can only be measured along the direction of the ultrasound beam, only a limited number of strain components can be measured by TDI. Usually, apical views are used to calculate the longitudinal strain and the parasternal short-axis views are used to calculate radial strain [14].

Conversely, STE is based on the detection on 2D images of the motion of acoustic markers (called “speckles”) generated by the interaction of ultrasounds with the myocardium. The position of the speckles can be tracked during the cardiac cycle by using specific software packages. The movement of speckles can be used to measure strain and calculate SR. To analyze the different components of myocardial deformation (strain) with STE is necessary to acquire several views: 4-chamber, 2-chamber, and apical long-axis views to compute global longitudinal strain (GLS) and short-axis views for circumferential and radial strains [15].

Strain and SR are used to estimate global and regional myocardial function. The main advantages of these parameters are (i) the relative independency by loading conditions as opposed to conventional chamber function parameters like ejection fraction (EF) or stroke volume and (ii) the ability to differentiate the active contraction of the myocardium from passive motion resulting from the global heart translation or from tethering by the surrounding myocardium [16].

Moreover, since it measures directly myocardial function, deformation imaging can detect subclinical myocardial dysfunction, when EF or other chamber function parameters are still in the normal range because the heart has activated its compensatory mechanisms. Since, during pregnancy, there is a continuous variation of the loading conditions of the heart, the use of STE can be particularly useful to study the changes occurring in the myocardial function during either normal or pathological pregnancy.

### 3.1. Strain: Basic Concepts

Strain measures the relative shortening/thickening of a myocardial segment during systole compared to its initial length/thickness in diastole and it is a parameter used in echocardiography to describe the myocardial deformation.

The linear strain (amount of deformation) can be defined by the formula

(1) $$\begin{array}{c} ɛ=\frac{\Delta L}{L0}, \end{array}$$

where ɛ is strain,* L*0 is baseline length, and Δ*L *is change in length.

Strain is unitless and it is expressed as a percentage. Strain can assume negative or positive values, which reflect shortening/thickening or lengthening/thinning, respectively [17].

**Figure 1:**
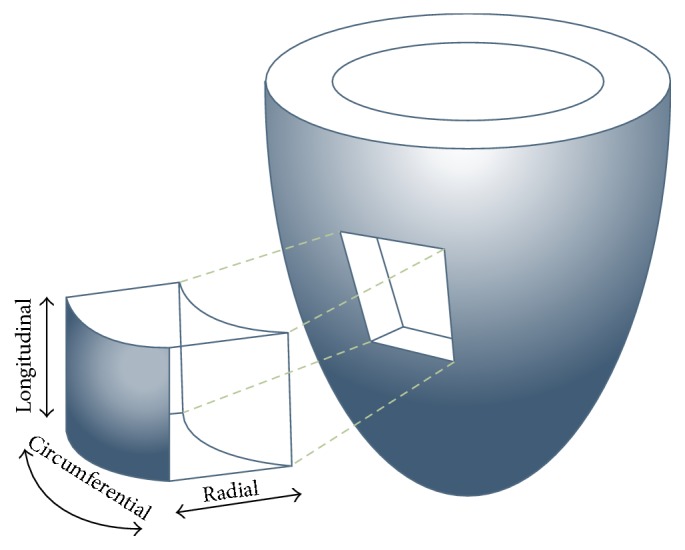
The image shows the three main components of myocardial deformation: longitudinal, radial, and circumferential.

**Figure 2:**
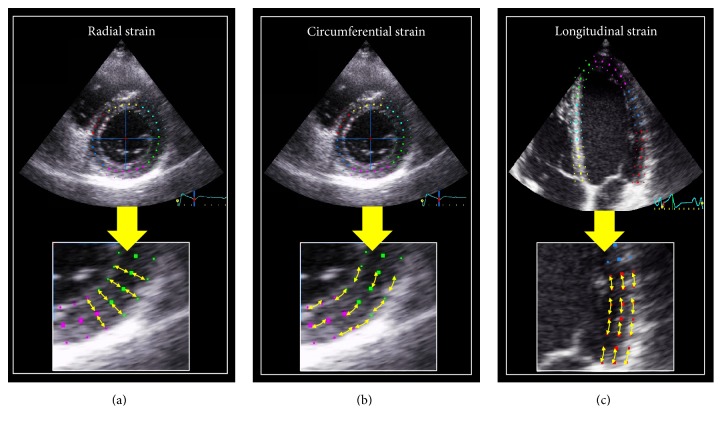
The figure shows speckle-tracking analysis and the main components of myocardial deformation: radial (a), circumferential (b), and longitudinal (c).

There are several components of myocardial deformation: longitudinal (measuring basal-apical shortening), radial (measuring myocardial thickening), and circumferential (measuring circular perimeter shortening) (Figures 1 and 2).

Since, during systole, the length of the LV shortens and its circular parameter decreases, the systolic values will be lower than diastolic ones. Accordingly, values for normal longitudinal and circumferential strain will be negative. Conversely, during systole, the thickness of myocardial segments increases. Therefore, the systolic values of radial strain will be higher than diastolic ones and values for normal radial strain will be positive [14]. SR describes the rate at which the myocardial deformation occurs and it is expressed as seconds^−1^ [18, 19]. Experimental studies have shown that the SR is less dependent on LV load variations and better reflects actual myocardial contractility than strain [20]. Nevertheless, the SR signal is noisier and less reproducible than strain; therefore, strain was the most frequently reported parameter in clinical studies assessing myocardial function. The utility of strain and SR has been documented in several clinical and experimental studies which showed that they can provide more information on pathophysiological mechanisms of cardiac dysfunction compared to conventional parameters describing cardiac chamber function (e.g., EF, stroke volume) [21, 22].

### 3.2. Strain Obtained from Tissue Doppler Imaging

The first echocardiographic method used to evaluate strain was TDI. By this method, SR was derived from the velocity data using the equation

(2) $$\begin{array}{c} SR=\frac{\left(V_{1}-V_{2}\right)}{L}, \end{array}$$

where *V*_1_ is the velocity at point 1, *V*_2_ is the velocity at point 2, and* L* is the distance between points 1 and 2, usually set at 10 mm. However, since it is a Doppler method, to avoid underestimation of velocities, it is essential that the ultrasound beam is aligned parallel to the direction of the myocardial wall motion being studied [19].

The myocardial velocities can be measured using either* spectral pulsed tissue Doppler (TVI PW) *or* 2-dimensional color-coded TDI image loop*. However, myocardial velocities obtained from the spectral pulsed TDI curves are higher than those from 2D color-coded TDI images, because the former measures peak velocities whereas the latter measures mean velocities [23].

It is important to take into account the fact that measurements performed by TDI may be influenced by both blood flow and movement of the other adjacent structures. Moreover, to minimize the effects of respiratory variations, the patient should be asked to suspend his/her breathing for several heartbeats and the operator should use respiratory maneuvers during acquisition to improve the quality of acquisitions [24].

### 3.3. Strain Obtained by Speckle-Tracking Echocardiography

**Figure 3:**
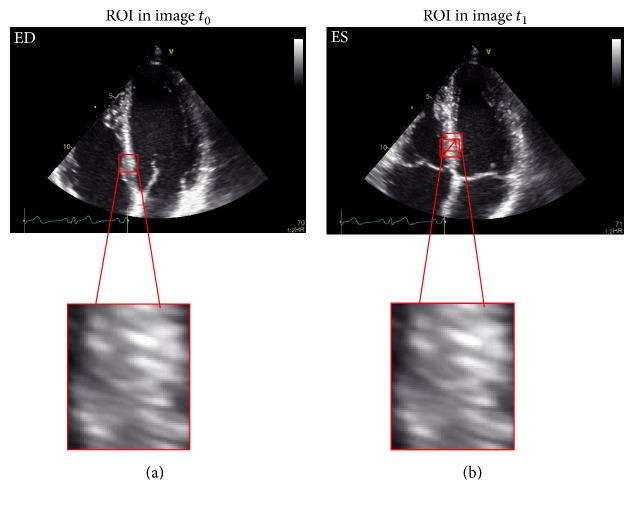
The image shows a region of interest (kernel) at *t*_0_ (a) and the relative change in its position at *t*_1_ (b). Myocardial speckles in the grayscale image are tracked frame-by-frame.

Speckle-tracking echocardiography is a relatively new ultrasound imaging technique based on the analysis of the spatial dislocation of spots generated by the interaction between the myocardial fibers and the ultrasound beam. These spots are defined as “speckles” which are merged in functional units (kernels) which have a unique pattern within the myocardium. Therefore, each kernel can be individually tracked during the cardiac cycle and its motion can be analyzed by dedicated software packages (Figure 3). By knowing the position of the kernels at the beginning and at the end of systole and the time between two frames (from frame rate), the software can automatically calculate the deformation of the myocardium (strain), the rate of the displacement, and the rate of the deformation (SR) [25].

This method, different from TDI, is angle-independent, provides accurate measures of myocardial deformation, and guarantees good intraobserver and interobserver reproducibility [26, 27]. GLS is a parameter of global LV function obtained by averaging the peak values of systolic strain obtained from the 17 segments of the LV [28]. Accordingly, to obtain GLS, it is necessary to acquire and analyze three apical views of the LV: 4-chamber, 2-chamber, and apical long axis.

A number of studies have demonstrated that GLS is an accurate parameter to evaluate myocardial function [29, 30]. It has been reported to be more sensitive than EF in identifying subclinical left ventricular dysfunction in cardiomyopathies [31] and has shown an independent prognostic value [32, 33]. Use of GLS to assess myocardial function has been recommended in a variety of clinical scenarios [15, 27, 34]. The reference values for STE have been included in the recent recommendations for cardiac chamber quantification by echocardiography in adults [35].

The strain and SR values obtained with TVI and STE are well correlated, even though the STE approach is more rapid and reproducible and allows a more complete evaluation of LV myocardial deformation due to its independence of the ultrasound beam alignment [26].

#### 3.3.1. 2D Speckle-Tracking Echocardiography Image Acquisition

To obtain images to be analyzed with the STE software package, it is recommended to optimize image quality by using a grayscale second-harmonic 2D imaging technique with careful adjustment of image contrast. The gain settings should be optimized, the depth should be reduced, and the focus should be in the middle of the left ventricle. Finally, images should have adequate temporal resolution (50–90 frames per second) [19]. Lower temporal resolutions will not allow a sufficient number of systolic frames to track the motion of the kernels. Higher temporal resolution will impact the spatial resolution of the images by reducing the number of scan lines [36]. Moreover, it is essential to optimize the LV border visualization. Care must be taken to avoid LV foreshortening and image acquisition should be performed during breath-hold to minimize respiratory interference. It is essential that the electrocardiographic trace is stable to avoid artefacts during the evaluation and at least three cardiac cycles should be acquired for each loop [37]. Artefacts, such as reverberation or shadowing, could affect strain computation and provide wrong strain values, which might erroneously suggest cardiac dysfunction.

#### 3.3.2. 2D Speckle-Tracking Echocardiography Image Analysis

The assessment of strain by 2D STE is a semiautomatic method. The images recorded are processed using dedicated software packages usually available on workstations or included in the echo machine. The apical long-axis view should be analyzed first to identify the end-systolic frame looking at the movement of the aortic valve. A pulsed-wave spectral Doppler envelope of LV outflow may be helpful to set the timing of cardiac events if the images of the aortic cusps are suboptimal [17].

Endocardial border is identified by the software in all apical views resulting in a manually adjustable region of interest, which should exclude the bright pericardium [38].

**Figure 4:**
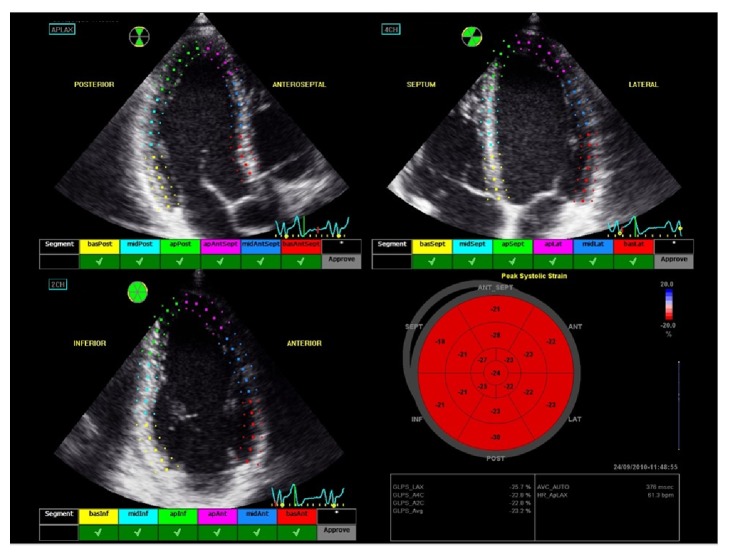
Regions of interest (dotted lines) have been traced on apical long axis, 4- and 2-chamber views. Each region of interest is divided into 6 segments for each view. The operator may accept/reject segments in case of suboptimal tracking of speckles.

The software package automatically divides each region of interest into 6 segments for each view and the operator may eventually accept/reject segments having the possibility of modifying the shape and the thickness of the region of interest in case of error of identification or incorrect tracking (Figure 4).

After operator approval of tracking, a deformation (strain) curve will be generated for each segment and the software package reports the peak value of strain occurring before aortic valve closure. GLS will be computed by averaging the segmental values of peak strain.

**Figure 5:**
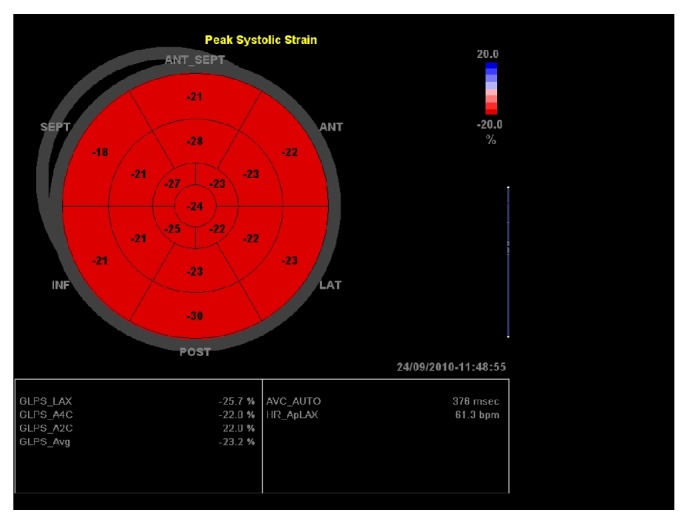
Bullseye display of segmental peak-systolic longitudinal strain. Global longitudinal strain is computed for each view.

To make interpretation of regional strain easier, the software generates a “bullseye” map with red and blue color coding, where the segments on the external ring represent the basal LV segments, the middle ring segments represent the midventricular segments, and the most internal ring represents the apical segments [39] (Figure 5).

Several independent studies confirmed that a peak GLS in the range of −20% can be expected in a healthy person [35].

A large study conducted on 247 healthy volunteers showed that longitudinal strain was significantly more negative in women than in men. Therefore, separate lower limits of normality for longitudinal strain should be used in men (−16.9%) and in women (−18.5%) [40].

#### 3.3.3. Three-Dimensional Speckle Tracking (3D STE)

2D STE has become a popular and clinically useful technique, but it is limited by the assumption that kernels move linearly along planes that coincide with the 2D echocardiographic view. Conversely, myocardial motion is quite complex and comprises rotation and displacement in the 3D space. Since using 2D STE might cause the loss of some kernels in the through-plane motion during the cardiac cycle, it has been suggested that 3D STE may be a more suitable tool to track the motion of the kernels in the 3D space [41].

To obtain myocardial deformation by 3D STE, we need a pyramidal full-volume data set of the LV that can be acquired using a matrix array transducer. To obtain a large data set which contains the whole LV with adequate temporal resolution (30–36 volumes per second), we need a multibeat acquisition to create a single volumetric data set by stitching together subvolumes obtained from 2 to 6 consecutive cardiac cycles [42]. To avoid the stitching artefacts, acquisitions should be done during suspended respiration and they are feasible only in patients with regular cardiac rhythm [43]. Since with 3D STE it is possible to follow kernels in a volumetric space, the software can calculate simultaneously the longitudinal, the radial, and the circumferential components of myocardial deformation, plus a composite parameter (area strain) which takes into account the simultaneous deformation in both longitudinal and circumferential direction at mid myocardium [44]. Data relating to the normal values in adult nonpregnant patients are known [45].

**Figure 6:**
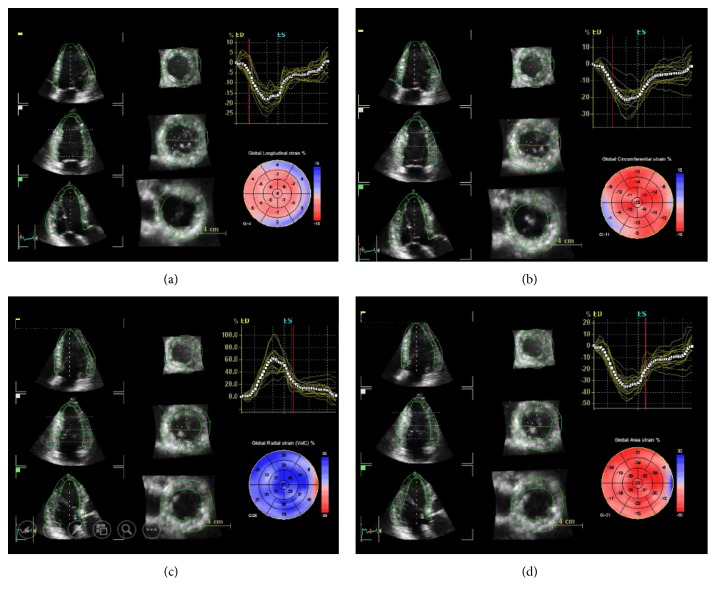
Three-dimensional speckle-tracking echocardiography. The software package calculates simultaneously the longitudinal (a), the circumferential (b), and the radial (c) components of myocardial deformation, plus a composite parameter (area strain) (d).

However, although it is theoretically appealing, at present, 3D STE still has several limitations that have not allowed for its wide use in daily clinical practice (Figure 6).

## 4. Clinical Applications of Deformation Imaging in Pathological Pregnancies

### 4.1. Preeclampsia

Preeclampsia is a hypertensive complication that affects 5–7% of pregnancies and is one of the most common causes of maternal morbidity and mortality [46]. In fact, it is considered a complex multiorgan disease potentially involving the kidney, liver, and cardiovascular and hematologic systems, as well as the brain. Autopsy data have shown a tenfold prevalence of myocardial contraction band necrosis in preeclamptic patients if compared with pregnant women that died from other causes [47]. Some studies have demonstrated persistent maternal cardiac impairment and hemodynamic changes years after delivery [48, 49]. Valensise et al. demonstrated that signs of left ventricular diastolic dysfunction and persistent heart remodeling persist in nonpregnant women before a second pregnancy with recurrent preeclampsia. These findings could raise an issue concerning preeclampsia as a cause or effect of heart remodeling [48–51].

The majority of published papers about echocardiographic assessment of maternal heart in preeclamptic women have used conventional parameters of cardiac function and remodeling [50–55]. In preeclamptic patients, the use of TDI and 2D STE demonstrated a reduction of both left and right ventricular diastolic and systolic function, also in preclinical stages of the disease, when cardiac output and EF are still preserved. LV-mass, LV-mass index, and LV wall thickness in preeclamptic women are higher than in healthy controls, reflecting the increase in LV afterload. Myocardial performance index (MPI), an index of reduced cardiac systolic and diastolic function, also increased. An impairment of right ventricular systolic function has been also described, reflecting the increase of pulmonary resistance secondary to LV diastolic dysfunction [56].

Moreover, several authors agree that longitudinal, radial, and circumferential strains are impaired in preeclampsia and may remain impaired also for months after the delivery, even in patients with preserved cardiac output and EF [48, 49, 55, 57, 58]. An interesting consideration is that coexisting LV hypertrophy and regional longitudinal systolic dysfunction could reflect a regional subendocardial impairment, probably due to subendocardial ischemia and/or fibrosis seen by Bauer et al. in preeclamptic women autopsies [47, 55]. Currently, new parameters based on 3D STE are emerging, showing an increased ability to detect subclinical myocardial impairment and early systolic and diastolic cardiac dysfunction. Myocardial dysfunction precedes chamber impairment and 3D STE can provide an assessment of global and regional LV function [59, 60]. Furthermore, some authors, using 3D STE, demonstrated that early onset preeclamptic patients presented worse cardiac remodeling than late onset preeclamptic patients, underlining the clinical relevance of detecting earlier and subtler cardiac dysfunction signs [58]. Other authors distinguish between preeclamptic and nonproteinuric hypertensive women, showing less impairment of longitudinal, circumferential, and radial strain in the latter [59]. These findings could mean that hypertension may not be the only cause of preeclamptic heart impairment, although women with nonproteinuric hypertension presented a worse cardiac function than healthy patients [57, 61].

### 4.2. Intrauterine Growth Restricted Pregnancies

Knowledge of heart disease in women is constantly evolving and emerging data show that complications of pregnancy such as preeclampsia and intrauterine growth restriction (IUGR) are predictors for the development of heart disease later in life [62]. Up to 10% of all pregnancies are affected by IUGR and its definition is controversial. The main reasons of IUGR are placental insufficiency and defective trophoblastic invasion, currently evaluated by the estimated fetal weight and umbilical artery Doppler flow velocity [63]. Fetal Doppler evaluation is a useful method to predict fetal compromise and permits distinguishing between severe IUGR and small for gestational age (SGA) fetuses [64]. However, different classifications are also reported in the literature and they could generate confusion in the definition of both maternal and fetal risk.

While maternal cardiac modifications occurring during normal pregnancy are well known, in normotensive pregnancies with IUGR, there are contradictory lines of evidence about maternal hemodynamics [3]. Some authors reported reduced cardiac output and left ventricular compliance [65], whereas others reported reduced maternal systolic cardiac function and increased total vascular resistance, without alterations of left diastolic function compared to physiological pregnancies [66].

Moreover, IUGR patients, compared with preeclamptic pregnancies, seem to present lower cardiac index, left ventricular diastolic dysfunction, and higher total vascular resistance index. Unlike preeclampsia, cardiac geometry and intrinsic myocardial contractility were reported to be preserved, but a third of IUGR patients present reduced diastolic reserve and an overt diastolic chamber dysfunction, despite a normal EF [67]. This suggests that the cardiovascular response is similar to that seen in preeclamptic patients, though less severe. Lack of physiological adaptation to the pregnancy, assimilating IUGR patients to a nonpregnant hemodynamic condition, could explain the reason of high resistance, low blood volume, and hypotensive condition, which characterized IUGR patient's condition [65]. The use of cardiac indices in isolation lies at the basis of several studies available in the literature, which make use of conventional 2D and Doppler transthoracic echocardiography [68]. A further improvement to the clinical presentation in IUGR patients could be gathered by including in the analysis the correlation between age and diastolic indices. The introduction of TDI and 2D STE techniques for analysis of myocardial deformation might allow an earlier diagnosis and better grading of cardiac dysfunction [69]. While several authors described the feasibility of STE in studying fetal heart function and morphology, in particular, the segmental and global systolic and diastolic velocities, strain, and SR values, few studies described its application for the evaluation of IUGR patients [70]. Krause et al. investigated maternal longitudinal mechanical dyssynchrony, a useful tool used for the evaluation of LV function, finding that pregnancies complicated by IUGR recorded significantly higher degrees of inter- and intraventricular dyssynchrony than those of normal controls [71].

## 5. Conclusion

Reduced maternal cardiac function in pregnancies that are complicated by preeclamptic and intrauterine growth restriction is the result of both reduced intrinsic myocardial contractility and reduced diastolic filling. Myocardial dysfunction can be present even in the presence of a normal ejection fraction, with significant decreases in radial, circumferential, and longitudinal strain values.

The use of 2D and 3D STE techniques to evaluate ventricular mechanics may help detect subclinical left ventricular dysfunction in women affected by obstetrical pathologies as preeclampsia and intrauterine growth restriction. Early detection of left ventricular dysfunction with the institution of appropriate treatment may reduce the risk of future CVD.

## Abbreviations

CVD: Cardiovascular disease
EF: Ejection fraction
GLS: Global longitudinal strain
LV: Left ventricle
SR: Strain rate
STE: Speckle-tracking echocardiography
3D STE: Three-dimensional speckle tracking.
